# The patient journey of newly arrived asylum seekers and responsiveness of care: A qualitative study in Germany

**DOI:** 10.1371/journal.pone.0270419

**Published:** 2022-06-24

**Authors:** Louise Biddle, Sandra Ziegler, Jenny Baron, Lea Flory, Kayvan Bozorgmehr

**Affiliations:** 1 Department of General Practice and Health Service Research, Section Health Equity Studies & Migration, University Hospital Heidelberg, Heidelberg, Germany; 2 Department of Population Medicine and Health Services Research, School of Public Health, Bielefeld University, Bielefeld, Germany; 3 Nationwide Working Group of Psychosocial Centres for Refugees and Victims of Torture e.V. (BAfF), Berlin, Germany; Public Library of Science, UNITED KINGDOM

## Abstract

**Background:**

Research on health and healthcare for asylum seekers and refugees (ASR) has focused strongly on accessibility and legal entitlements, with quality of care receiving little attention. This study aimed to assess responsiveness, as non-medical quality of care, in the narratives of ASR patients recently arrived in Germany.

**Methods:**

31 ASR with existing medical conditions were recruited in six refugee reception centres and three psychosocial centres. Semi-structured, qualitative interviews were conducted which reconstructed their patient journey after arrival in Germany. Interviews were recorded, transcribed verbatim and evaluated using thematic analysis.

**Results:**

The experiences of participants throughout the patient journey provided a rich and varied description of the responsiveness of health services. Some dimensions of responsiveness, including respectful treatment, clear communication and trust, resurfaced throughout the narratives. These factors were prominent reasons for positive evaluations of the health system, and negative experiences were reported in their absence. Other dimensions, including cleanliness of facilities, autonomy of decision-making and choice of provider were raised seldomly. Positive experiences in Germany were often set in contrast to negative experiences in the participants’ countries of origin or during transit. Furthermore, many participants evaluated their experience with healthcare services in terms of the perceived technical quality of medical care rather than with reference to responsiveness.

**Conclusion:**

This qualitative study among ASR analysed patient experiences to better understand responsiveness of care for this population. While our results show high overall satisfaction with health services in Germany, using the lens of responsiveness allowed us to identify particular policy areas where care can be strengthened further. These include in particular the expansion of high-quality interpreting services, provision of professional training to increase the competency of healthcare staff in caring for a diverse patient population, as well as an alignment between healthcare and asylum processes to promote continuity of care.

## 1. Introduction

Asylum seekers and refugees (ASR) are exposed to a number of risk factors before, during and after migration which decrease their chances of mental and physical wellbeing. The UCL-Lancet commission on migration and health reports that mental health, chronic illnesses, and serious infectious diseases are issues which need to be addressed in particular for newly arriving ASR [1]. It is therefore essential that health systems in resettlement countries provide the required services for ASR to aid their recovery and integration in host communities. Since health care access for ASR is restricted in many European countries [2], a focus of public health scientists and activists has been on issues of legal entitlements and accessibility of health services [3–6].

Although access to services is crucial, the quality of health care that ASR patients receive deserves the same attention. Even when legal access is granted, other barriers such as language difficulties or discriminatory practices can pose obstacles to high quality health care [7]. Therefore, non-medical aspects of the quality of care, namely responsiveness, should receive more attention. Responsiveness is defined as the ability of the system to respond to the “legitimate expectations of the population” regarding non-health enhancing aspects of care [8]. Responsiveness refers to “respect for persons” and “client orientation” aspects of the doctor-patient interaction [9]. Meeting the corresponding expectations in healthcare interactions is crucial in fostering a positive healthcare relationship and ensuring the continuation of care: patients who have had bad experiences in the healthcare encounter are less likely to adhere to the doctor’s recommendations, take the prescribed medicines, or continue to engage with healthcare services [10, 11].

In Germany, encounters with the health system for ASR are shaped by multiple providers and professionals depending on the time since arrival, asylum status and type of accommodation [12]. Upon arrival, refugees are housed in large, collective reception centres with shared sleeping and sanitary arrangements. During this time, the responsibility for organising and financing medical care lies with the state authorities. Many reception centres have on-site outpatient clinics which, in addition to providing low-threshold primary care services, may offer specialist surgeries offered by gynaecologists, psychologists or paediatricians depending on local arrangements [13]. Sometimes, such clinics will work with a pool of interpreters to aid communication. If treatment is required that cannot be covered locally, patients are referred to off-site specialists or hospitals. However, medical care for refugees is restricted by law to treatment for “acute illness and pain” [14] during the first 18 months after arrival in Germany or until a positive asylum decision. Other services that are deemed “essential” can be requested on a case-by-case basis [15]. In the majority of federal states, health services can only be accessed via a healthcare voucher, which has to be requested at the local social security office. This is a bureaucratic process which has been documented to delay care substantially [16–18].

ASR are often transferred from the initial reception centre to other reception centres within the federal state once their asylum claim has been lodged. Individuals from so-called “safe” countries of origin usually remain in reception centres until a decision has been made on their asylum claim [19], but others may be transferred further to smaller, decentralised accommodation centres. Here, the responsibility for medical care lies with the regional authorities, and primary care is not usually provided within the accommodation. Depending on the local circumstances, they may encounter challenges such as the physical accessibility of services in remote areas [20]. The transfer of medical information between reception centres, but also into the community, has also been reported as a challenge to adequate care as medical documentation systems and electronic health records are fragmented and usually not mutually compatible [21].

Thus, the patient journey of ASR in the German health system is shaped by complex rules and procedures. ASR may encounter multiple different healthcare providers during their first months in Germany and face legal and structural barriers in accessing and receiving adequate care. Existing studies on experiences of ASR patients in Germany find that access to health care is perceived as “highly hurdled, bureaucratically inefficient, and disempowering” [22]. Language barriers are reported as being a major obstacle, impeding access to medicines and shared decision-making [23]. One study concludes that quality of care needs to be improved, especially for non-english speaking patients and female ASR [24]. On the other hand, medical service encounters were perceived as satisfying and engaging [22, 24]. However, so far little research has considered how patients experience and evaluate the medical interaction in this unique context throughout the entire patient journey using a structured assessment framework, covering patient encounters with a multitude of different providers which make up the overall experience with the health system. Such an assessment is required in order to identify priorities for the improvement of care for ASR patients.

The present analysis uses the lens of responsiveness to shed light on patient experiences and to assess the non-medical aspects of healthcare provision for ASR in Germany. We first aim to identify elements of responsiveness which are deemed important by ASR when recounting their patient journey. Second, we aim to understand which attributes of the care environment may be supporting or impinging on these elements from the patient perspective.

## 2. Methods

This study was designed as a semi-structured, qualitative interview study with ASR with existing illnesses or health problems. The interviews were intended to capture participants’ patient journeys after arrival in Germany, allowing for personal narratives about living with illness throughout the asylum process, including experiences prior to, during and after migration to emerge. The semi-structured interview schedule did not probe directly for responsiveness domains, but let them emerge naturally. It included prompts for the first contact with healthcare services in Germany, experiences with medical encounters and care processes, comparisons of care in different settings, support needs and wishes for the future. Space was given for narratives about health and health care experiences in the pre- or peri-migration stage if participants wanted to share these.

Ethical clearance for the study was obtained from the Ethics committee of Heidelberg University Hospital (S-287/2017). Participants were recruited via healthcare professionals in six outpatient clinics in reception centres (RC; 25 participants) and 3 non-governmental psychosocial centres for refugees and victims of torture (PSC; 6 participants). Criteria for inclusion in the study was the presence of an existing chronic illness, disability, mental health issue or serious infectious disease. Participants were informed about the aims and purpose of the study by the healthcare provider and, if interested, put in contact with a member of the research team to schedule a personal meeting. During the personal meeting, aims, purpose, handling of data and data protection were provided verbally and in writing in a language the participant could understand–either by the researcher or through a professional interpreter. Full written consent was obtained from all participants prior to the start of the interview.

Interviews were conducted from August 2017 to April 2018 by SZ, LB, JB, LF and a fifth female researcher (FR) and the majority of interviews were conducted by two researchers. All interviewers had experience in conducting qualitative research and had worked in the context of healthcare provision for ASR previously. Interviewers’ professional backgrounds included public health (LB), medicine (FR), social and cultural anthropology (SZ) and psychology (JB, LF). During the research process, interviewers were teamed up in inter-professional teams for the conduct of the interviews to maximise the benefits of different professional perspectives. Interviews were carried out in a private room–either in a residential facility, medical clinic or psychosocial centre–to protect the confidentiality of participants, although four participants chose to have their husband, wife or children present during the interview. Water, juice and snacks were provided to create a welcoming and relaxed atmosphere. Interviews in which the chosen language was either English or German were conducted by the researchers. For other languages, an on-demand video-interpreting service was used to carry out the interviews in reception centres, which was chosen on the basis of working with trained and certified interpreters and complying with the data protection requirements of the study. For interviews in the psychosocial centres, interpreters were present in person. Interpreters were informed about aims of the study upfront and provided with the interview guide. They were asked to translate in the first-person form, however, sometimes maintaining this was a challenge during the course of the interview. Interviews lasted between 16 and 109 minutes and were voice-recorded digitally. All researchers kept field notes to document the circumstances of each interview, non-verbal cues and subjective impressions as well as conversations arising immediately after the interviews.

For analysis, interview recordings were transcribed verbatim using f4 transcription software. Analysis was conducted by LB and SZ using a qualitative content analysis methodology [25]. Both researchers read the transcripts to familiarise themselves thoroughly with the interviews. An initial coding system was devised based on the interview schedule, field notes and initial impressions of the interviews. This was divided into the main stages of the patient journey in medical sectors: mandatory health examination, contact with the outpatient clinic in reception centres, appointments with medical specialists and psychologists, inpatient stays in hospitals and transfer to different accommodation facilities. Within each sector, separate codes covered descriptions of the care processes, positive and negative experiences, aspects of communication and health literacy as well as reflections regarding the relationship with medical personnel. The code system also included additional topics which did not neatly fit the “patient journey” structure, including aspects of the asylum process, the living environment, social networks, financing of care and reports of equality or discrimination related to health care. An “other” category was included within each overarching theme to capture additional emerging themes. The initial coding framework was tested on four interview transcripts by both researchers concurrently, after which the framework was revised and refined (see S3 File for coding framework). The remaining transcripts were then coded separately by LB and SZ. Following the completion of the coding process, analysis of codes was conducted in two steps. First, each of the two researchers were assigned specific thematic areas and produced a descriptive summary of the content of the interviews within each area, including anchor quotes showing the full continuum of responses. Each section was then cross-checked and discussed with the other researcher and subsequently refined with all authors. In a second step, the initial summary of the patient journey was assessed for the presence and influence of the eight domains of the WHO responsiveness framework [10] (see Table 1). The question was if and how these topics where thematised within the narratives of patient experiences of ASR.

**Table 1:** Responsiveness domains (WHO 2000).

Dignity	Respectful treatment
**Confidentiality**	Protecting personal information and privacy
**Autonomy**	Informed, shared decision-making
**Communication**	Understandable information, interactive discussion
**Prompt Attention**	Accessibility, reasonable waiting times
**Choice of Provider**	Choosing facility, specialist or specific physician
**Basic Amenities**	e.g., cleanliness of facilities
**Provision of Social Needs**	Access to networks, social support

Enshrined as one of the key outcomes of well-functioning health systems by the World Health Organization (WHO) in the World Health Report 2000 [26], the concept of responsiveness encompasses eight domains: respectful treatment, prompt attention, communication, quality of basic amenities, confidentiality, choice, social support and autonomy. In sum, these are intended to capture the non-medical aspects of the healthcare encounter as they relate to the specific dimensions considered vital for a successful medical interaction [10]. We additionally considered the dimension of “trust” as important for the responsiveness of care, as suggested by Röttger et al. [27]. In line with existing theoretical literature, we understand “trust” as the optimistic acceptance of an uncertain situation, predicting that the trustee will act in the truster’s interests.”[28] The analysis of the responsiveness domains was carried out in several joint discussions between LB and SZ, with the continued involvement of all authors. For analysis and reporting, all place and personal names were replaced with fictional ones.

## 3. Results

### 3.1 Description of participants

A total of 31 ASR were interviewed; 18 males and 13 females (see S1 File Attachment 1 for details of participants). 24 of the interviews were carried out with only one participant, but two interviews were conducted with married couples, where both partners had health issues, and one interview was conducted with a group of three women, by their own request. Ages ranged between 20 and 65 years, with a mean age of 39 years. The sample included 18 different nationalities, 11 from European, 11 from African and nine from Asian countries. 14 participants had been resident in Germany for less than six months, with two participants having arrived just a week prior to the interview. Within the group recruited in reception centres, the maximum duration of stay in Germany was 18 months. 16 of the participants had an ongoing asylum process, seven had their asylum claim rejected while five were granted a temporary right to stay (“Duldung”). The six participants recruited in the psychosocial centres had been resident in Germany between two to 18 years, three of them had a secured asylum status (S1 File Attachment 1).

With regard to health issues, 19 participants cited mental health issues as one of their primary health concerns. Chronic illnesses were also common, including hypertension (3 participants), diabetes (3 participants), as well as chronic heart failure, epilepsy, morbus crohn, chronic kidney disease and myasthenia gravis (1 participant each). Two participants had a serious infectious disease: one HIV and the other tuberculosis (see S1 File Attachment 1).

### 3.2 Mandatory health examination in Germany

The first point of contact with the medical system for ASR upon arrival in Germany is the initial health examination; a medical check-up, vaccination and x-ray which is mandatory for all new arrivals. However, none of the interviewees retrospectively regarded this experience as their first encounter with the health system. They only elaborated on this when asked about it specifically, mentioning mostly the x-ray and vaccination. Several participants regarded the procedure as routine and “absolutely normal” (ASR_18E) and did not further question it, as so many other asylum seekers went through it.

Asked about possible reasons for such an examination, several participants declared that it was for their own protection and that of others and that they **trusted** the government to do what is right:

*Interviewer*: *What was the vaccination about?*

*ASR_14D: I don’t know. Me, I cannot question the Government but I know it’s for our good*. *I know maybe their vaccine is to protect us from some Illnesses. It’s for our good. Yeah, I know.*

Although experiences with the health examination were generally described as positive or neutral, this participant mentioned **communication and information** deficits, partly due to a lack of translators available:

*Interviewer*: *And did they explain to you why they are doing this check?*

*ASR_18E: How could they because we can’t speak German*, *they can’t speak Asmait that was just routine work for them and I do understand that.*

In one case, the absence of translators led to a misunderstanding between the physician of the public health office and the refugee, leading the latter to believe the vaccinations were mandatory, when in fact they were voluntary. Even in this case of impaired autonomy the newly arrived refugee **trusted** the physician to know what they were doing.

*“We stood there, waiting for a long time*. *We did not even know what was happening. It was all really unpleasant. You know, we are in a foreign country now. And we also didn’t know that we had the choice not to do it. But we, so to say, trusted them, that they know what they are doing.” (ASR_4.2B)*

### 3.3 Primary care clinics in reception centres

While living in reception centres, participants reported that they could usually find someone to contact with any urgent medical problem–either in the on-site outpatient clinic during opening hours or by turning to security or accommodation management personnel, which was able to promptly call a taxi or an ambulance to take them to the nearest hospital.

Outpatient clinics are staffed by physicians and nurses who provide primary care, sometimes supplemented by specialist surgeries. However, since the walk-in clinics provide low-threshold care without appointments, several participants reported long **waiting times** during the general opening hours. This was the case particularly in an initial reception centre of the federal state where there was insufficient capacity to meet the demands of the many asylum seekers which arrived daily. One patient reported he did not return there after having been turned away several times.

*“*Each time when I go there they say […] just come […] tomorrow*, *I go and come [the next morning] they will say two o’clock*, *they say "No*! *come at two after lunch" when I go there they will say "No*! *There’s just one doctor*, *people are too many*. *[…] All those things*…*I get fed up” (ASR_16D)*.*

The waiting times in subsequent reception centres were deemed to be more acceptable (ASR_11D, ASR_23F).

Participants generally rated the primary care clinics positively, but specific reasons were only given when prompted. Medical care was rated positively when healthcare staff was deemed to be medically competent, professional and “caring”:

*[…] they take care of me*. *Honestly everything is just perfect for my own, for me because since I’ve been here they are taking care of me, yeah. (ASR_3A)*.

[…] *I have to say the doctor acted professionally according to his medical expertise and also looked out for everything* (*ASR_21F)*

An improvement in health, or a sincere engagement of the medical staff to this end, had a positive impact on the assessment of care by patients:

*They are very good*. *Each time I went to them they will give me medicine once I complain that "this is my problem*, *I feel pain*, *I feel headache*…*" they give me medicine they are okay*. (*ASR_3A)*.

[…] *they are really trying, the way I came has improved better than the way I used to be, that’s my own experience (ASR_6C)*

Several participants specifically reported that this “care” extended beyond what they expected from regular medical care to encompass their specific needs. In one case, doctors recognised the needs for additional psychotherapeutic support for a participant, even though he himself had not expressed this (ASR_15D). In another case, physicians conducted house visits when the mobility of their patient was limited (ASR_3A). “Caring” may even extend beyond medical concerns, for example when a nurse, after booking an appointment with a specialist in town, printed out directions and explained how to get there (ASR_20F, ASR_18E). When health care providers took the particular circumstances of the patient into account, we interpreted this “caring attitude” as a mark of **respect**, as they take a holistic view of the situation and needs of their patients and ensure they have everything they need in order to get better.

A further mark of **respect** was when patients felt “taken seriously” by physicians. One patient was particularly moved by the fact that his medical concerns were being taken seriously in the clinic, especially since he did not previously have this experience in his home country:

*[It was] good that they believed me, that I am sick. They always wrote to [the authorities]: This man is sick. He needs help, you need to support him and so forth*. *In my home country this never happens. >cries< In my home country I was in hospital once, they sent me home. I was bleeding too badly. They said, it’s not that bad. (ASR_11D)*

Another patient compared different experiences of being taken seriously within the outpatient clinics of different facilities.

*Yeah in Muldau I tried to explain to them but they were not taking it so serious like the people here in Friesenberg. [When my afflictions started] it was giving me headache so in Muldau they were just giving me Ibuprofen*…*Ibuprofen, Ibuprofen… They didn’t send me to the hospital.” (ASR_16D)*

ASR may be transferred between multiple reception centres and therefore from one care structure to another. It may be in some part for this reason that the **relationship** between patients and medical staff was rarely the focus of refugees’ patient journey reflections, since the establishment of a such relationships takes time. One participant rated his relationship with the medical staff as very positive after seven months of living in that particular setting (ASR_2A), another participant said it took several meetings with the doctors until they were able to judge each other (ASR_12D). This might be the reason why questions about the doctor-patient relationship often resulted in relatively short responses which referenced the medical competency of physicians. Physicians were imbued with **trust** because of their profession and the assumed competency which it entails: *„I trust the doctors*, *the doctors are good*.*”(ASR_2A)*. Asked for relational aspects of the doctor-patient encounters, physicians were oftentimes simply described as doing-their-job; listening to and understanding their patients was considered part of that job: *„you’re welcome to explain them your problem*, *they are asking*, *they must listening to you” (ASR_12D)*

However, a patient-centred and empathic **communication** influenced refugees’ assessment of care. Taking time to listen to patients and understand their concerns was viewed positively, for example referring to a psychologist:

*She now listen*. *I talk and cry and so she have many many—patience to listen and every time have something nice to tell me that I think ok be better.” (ASR_20F)*

On the flip side, if physicians did not take the time to listen to patient concerns this is raised as a point of potential dissatisfaction: *“What they lack here is the doctors that are working here they don’t listen*. *[…] They don’t listen at all*.*” (ASR_6C)*

Successful communication between patients and medical staff was highly dependent on the linguistic capabilities of both parties. Although communication in English was possible with many physicians (ASR_17D, ASR_3A, ASR_12D), participants who did not speak English felt that this impaired the establishment of a relationship with healthcare professionals (AS2_KA). Whether clinics had interpreters on hand and what languages were covered differed considerably between the reception centres. Some participants even reported communication problems if interpreters were provided by outpatient clinics, due to different dialects or the poor quality of the translation:

*There is a Sorani interpreter but that is a different dialect that I don’t understand so well, and he is also not always there*. *Then there’s an Arabic interpreter but he’s from Algeria and […] they also have a specific arabic dialect […] that’s very difficult for me to understand. (ASR_8C)*

*If there were interpreters then they were very bad […] a really low standard. And after that of course it was the case that the doctor, that I didn’t understand, so the communication didn’t work*. *And then of course there was irritation and they were annoyed […] because of the papers and the secretariat and these things.”(ASR_21F)*

Many participants reported asking friends (ASR_22F), other refugees (ASR_27PSC) or family members (ASR_19E) to act as interpreters at medical appointments. Even members of staff in the clinic, including security personnel, helped out to translate if *“there is no alternative”* (ASR_19E). Participants consistently reported being grateful for this kind of unpaid support.

### 3.4 Appointments with specialists

Patients can be referred to specialist providers outside the reception centre if required. In total, seven participants had experiences with visits to specialist physicians. This included appointments with ophthamologists, orthopedics, dermatologists, neurologists, psychiatrists and otorhinolaryngologists. Ten further participants reported having visited or currently being in treatment with a psychologist–although participants did not always make a clear distinction between psychologists and psychiatrists. For the purposes of this analysis, treatment by specialist physicians and psychologists will be considered jointly.

Technically, refugees have free **choice** of their provider in Germany provided they have received a healthcare voucher. However, due to the limited knowledge of newly arrived refugees of the German health system and of the possibly limited availability of providers in the area, general practitioners in refugee outpatient clinics may arrange appointments with particular specialists on the grounds of a previous cooperation or because of certain language competencies. Our participants recognised the function of the primary care clinic as a gatekeeper:

*“In Hoffingen I went to […] a doctor who then referred me on to a psychotherapist*. *[…] That’s just the typical procedure, you can’t just go directly to the psychotherapist” (ASR_18E).*

Some participants reported that they had to **wait for a long time** before the appointment with the specialist:

“*Now I go to [the refugee outpatient clinic] they say there’s many people in Germany in Fürstingen who have this problem and they don’t*…*There’s no more […] appointment*…*”* (*ASR_15D)*.

The issue of timely appointments was raised in particular regarding treatment for psychological issues. However, the assessment of what “timely” entails varied among the participants. While one participant was disappointed that his appointment with a psychologist could only be made after 20 days (ASR_8C), another participant reported that she did not receive any care through regular care structures and waited 5 years before she received treatment in a psychosocial centre (ASR_27PSC). A further participant, however, reported no problems receiving timely treatment through a psychosocial centre in a different location (ASR_25PSC).

Ensuring adequate translation during a specialist appointment was often the responsibility of the patient (ASR_8C, ASR_21F), as interpreting services were not generally provided. Participants reported taking friends and family, as well as other refugees, along to the appointment to aid **communication**. However, finding someone to come along to the specialist could be a challenging task:

*I just asked the people here in the camp myself if they could translate for me, but that was quite difficult. One guy could translate but he has a family and didn’t have time*. *Another could speak Arabic and with Arabic it’s not so easy, I can’t understand that very well. In the end I came to this guy who could speak English and we agreed that he would accompany me and translate for me (ASR_8C).*

The informal nature of such interpretation arrangements could lead to substantial problems in the care process, including cancelled appointments due to a lack of interpreters (ASR_21F), a perceived lack of an adequate treatment plan (ASR_8C) and misunderstandings during the appointment:

*The friend who translated for me talked in English, but the doctor then answered in German, which is why we don’t know quite what he said or how he responded*. *But I think that the doctor still broadly understood what my problem was, but what he wanted to communicate to me I did not understand. (ASR_8C)*

After the visit to the specialist, some participants reported not knowing the results and consequences of their consultation. This seemed not always to be related to language barriers. One participant reported a lack of patient information on the side of the specialists towards him and the primary care clinic to ensure continuity of care:

*He neither told me anything nor did he give me a prescription*. *I was examined, but I didn’t receive any kind of document. I don’t know now if everything is okay or not. I was referred there because of my high blood pressure, and yes of course he did a thorough examination, checked whether my heart was okay, […] but otherwise there was no result. (ASR_18E)*

Experiences and assessments of the doctor-patient interaction were largely related to the medical competency of the doctors and the improvement of the medical condition that gave reason for the consultation:

*Interviewer*: *[…] How was your experience with the dentist?*

*ASR_18E*: *Super*

*Interviewer*: *Why?*

*ASR_18E*: *I had a tooth ache, then they pulled the tooth now I have no more problems.*

A notable exception to this recurring form of reporting and evaluation were appointments made with psychologists, psychotherapists or psychiatrists. Here references to a positive relationship that was fostered through **respectful communication** were made:

*When we talk he asks me how I am, if I can sleep at night, if I am scared, if I am depressed and I answer him. That’s the course of our conversation*. *As a human he is really very valuable and a very good person, he takes good care of his patients and leads very good conversations with them, so I can only praise him. (ASR_18E)*

A **trusting** relationship with mental health specialists could be developed through continued engagement. In particular, the psychosocial centres were described as familiar places in which trusting relationships with psychologists could be fostered:

*I feel very calm here, maybe because I’ve known this place for so long*. *[If] I go somewhere else then I can’t even talk with the other doctors, and here I can talk about whatever I want (ASR_24PSC)*

Like in other care sectors, if an interest was taken in aspects of the patient’s life which influenced their illness and treatment this further supported a **trusting** doctor-patient relationship. One patient reported that she was given not only psychological support during her visits to the psychosocial centre, but also support in other administrative issues of everyday life:

*In different issues where she needed support*, *with some documents*, *or relating to the school or other things*. *[…] That really helped her*, *that’s why she feels so sheltered here*, *so safe*. *(Interpreter of ASR_25PSC*)

The lack of involvement of medical staff in the asylum case of their patients, can, in turn, also negatively affect the doctor-patient interaction. As medical documents are sometimes requested by patients or their lawyers to support an asylum case, for example to prevent a deportation on medical grounds, the refusal to provide the required documents or the inability to provide medically justified information that helps the case may lead to a break of **trust** between doctor and patient. Particularly in the case of psychological treatment, which relies on a trusting relationship, such an event may negatively affect further treatment, as is exemplified by one participant who interpreted the failure of his doctor to “help out” with the right documents as a personal affront and unethical professional behaviour:

*I talked to him and said: “Why did you write such a certificate? It’s not sufficient for me!” “Yes, unfortunately I can’t do that.” “Why can’t you do that? You know exactly what kind of problems I have*.” *He knows about my panic attacks and everything. He promised me, if you need help, come to me and still. […] I always ask: Imagine I am a doctor and you are the patient. And you have problems. Why should I not help you? If I see that you are suffering and you have this and you have that. And you tell me so many things. I only watch and blablabla. Good Bye! Why then am I a doctor. Go work at the construction site then and don’t be a doctor! What the doctor there for? To help people. Especially a psychologist. (ASR_11D)*

### 3.5 Experiences in the hospital

14 participants reported having had experiences in a German hospital, with nine staying as an inpatient. Some were referred for a scheduled inpatient treatment, while others went to the emergency room. Three participants reported having been brought to the hospital in an ambulance.

In general, health care in German hospitals was evaluated positively. Medical staff would ensure all the right diagnostic tests were done (ASR_14D), followed all proper medical procedure (ASR_10C), took care that the patients had everything they needed to ensure a speedy recovery (ASR_13D) and would only release patients from the hospital if there were no outstanding issues (ASR_7.1C).

Being treated in a **respectful and friendly** manner was linked to a positive assessment of the hospital stay, pointing to the importance of the interpersonal level of care:

*On the first day I was a bit unsure*, *but then I felt wonderful because the people were so friendly, so kind and they always asked me what I wanted, if I want tea, if I want coffee or if I need anything. I thought that was wonderful.” (ASR_5B)*

Like in the setting of the primary care clinics, the “caring” attitude of staff was commended in particular. One participant noted that the nursing staff always asked him how he was doing, if he was taking his medication and encouraging him to get out of bed (ASR_14D).

Many participants displayed a great degree of upfront **trust** in their doctors. For some participants, this trust seemed to not require particularly positive experiences, but was granted on the basis of their profession.

*Interviewer*: *And you say, the doctors prescribed these tablets and made decisions. Did you trust the doctors, that he will do the right thing for you?*

*Interpreter of ASR_19E*: *Yes, everything was fine. And she had this trust, because the doctors know what they are doing*

Yet, for another participant, the degree of **trust** conferred in his doctor was related to the number of previous interactions:

*The doctors in the hospital? I won’t say trust him but the fifth time I was there I trust them, they are good. (ASR_3A)*

However, the frequent rotations of doctors in the hospital may have impeded the establishment of a trusting relationship. One participant therefore refused to talk to any of the other doctors, except for the one he established a relationship with first:

*I have to have one case with one doctor, if the other one comes then we won’t discuss with the other one*, *I will wait until the first one. (AS5_SI)*

Some participants explicitly mentioned that the medical care provided in the hospital exceeded their expectations. For example, when doctors removed two polyps during an endoscopy which was being done for different reasons:

*I once went to the hospital for a colonoscopy*. *I was examined, under narcosis. And bang, they got out two polyps. I didn’t know. (ASR_11D)*

However, a perceived lack of thorough care also characterised particularly negative experiences in the hospital. In particular, a disregard for the person and their broader situation–which we consider being a mark of **disrespect**–was criticised. The husband of one of our participants described that his wife was discharged from hospital too quickly, without regard for her mental state:

*They woke her up very early, at 5am, handed her some papers and she had to go*. *And she said, she has to wait for her husband. And they said: “No, you have to leave now.” And then she didn’t know where she should go and she didn’t remember where she was. […] She got onto the tram, she didn’t know where she was going. After a few stops she got out and tried to ask where this address was, how she can get there. And then an acquaintance found her and explained where she should go.” (Report of the husband of ASR_19E)*

Another patient reported a negative hospital experience where she was not taken seriously by medical staff, as a result of which she avoided hospitals altogether.

*She complained and said "I feel dizzy and I don’t feel well, and when I stand I faint" and things like that, yes, so it went back and forth, she said "(I can’t), please go home again", and since then she said*: *"No matter what happens to me I don’t want to go to the hospital so [quickly], because they haven’t really noticed my complaint". (Interpreter of ASR_26PSC)*

Focussing on hospital structures, some patients reported long **waiting times,** for example in the emergency room (ASR_20F). One participant saw the waiting as part of his patient duty for free treatment:

*[…] it’s not my money I’m pay, you know*? *[…] See you have to respect you have to wait, you know? (ASR_12D).*

Another participant explained that the waiting time may have been a result of the high workload of medical staff, who have other patients to take care of and therefore need to prioritise:

*Maybe they will spend more time on somebody, maybe I don’t know why they are spending*, *but as a human being [I] will not be too comfortable to say "Why you just walk pass on my bed?” (ASR_16D)*

While some waiting arrangements were understood, other processes and procedures occurring within the hospital stayed intransparent and were therefore not comprehensible for patients, suggesting a lack of adequate **communication.** For example, one participant did not understand why she had to remain in the hospital while the doctors were waiting for the results of diagnostic tests:

*Then I have to go to Camp maybe once you get the results you can let me know […] because I can’t wait until*, *I can’t sleep in this hospital till the results is out. (ASR_3A).*

Another participant complained that doctors did not adequately pass on information between rotations, requesting information multiple times and giving different recommendations on the medication she should take:

*[…] the next day the second come, different case entirely, he doesn’t even know what I discussed with the other one*, *I was angry. Even the drugs, some of them they are doctors and they don’t even know the drugs […] I am taking. Some don’t know, this one say stop, the other one don’t know I am taking, the other one said stop (ASR_6C).*

Some participants also pointed to a **lack of patient information and communication** during their hospital stay. One interviewee was surprised that he was *“completely gone”* during the implantation of his cardiac pacemaker, when he was only expecting a local anaesthetic (ASR_5B). Another participant reported that the way in which his medication worked was not really explained to him, although his discharge from the psychiatric hospital depended on him taking it:

*The medication is a kind of sedative. The ones are so you can sleep better, the others, well I assume they’re all kind of sedatives*. *[…] It wasn’t explained in detail, every medication the effect and what exactly they are for […] (ASR_9C)*

Just as in other settings, **communication** between patients and medical staff can be complicated by language barriers. For some participants, communication was possible in English (ASR_6C, ASR_20F), while others reported that the medical staff only spoke German, which made communication difficult but not impossible:

*Always German, [if] they speak slowly I understand everything but [if] they speak fast I don*’*t understand. So slowly, slowly I understand everything prefect, no problem (ASR_13D)*

If patients spoke neither English nor German, however, this impeded their access to adequate care (ASR_21F, ASR_22F). One participant suspected he would have been admitted as an inpatient if he had been able to communicate with the doctors:

*I know that I received a referral from the doctor in the camp, so that I will be admitted to hospital as an inpatient*. *I had to take my things with me, so I could stay in the hospital. A friend of mine came with me but he did not understand enough, did not speak good German and then, when I was in the hospital, all they did was an ultrasound and then discharged me home. I think, but I didn’t understand this well, it might have been because I didn’t have translator. I suspect that might have been the reason but I’m not sure.” (ASR_22F)*

Like in other settings, other patients (ASR_15D) or healthcare staff (ASR_5B) sometimes helped out as interpreters. Even basic knowledge of other languages was put to use, as in one case where a participant described accompanying another asylum seeker for her appendicitis and translating in English, even though his mother tongue was French (ASR_5B).

### 3.6 General assessment of health care and the healthcare system

#### 3.6.1 Perceptions of care restrictions and interpersonal discrimination

Regardless of the sectors in which healthcare is provided, access to health services is restricted during the first 18 month of residency in Germany. Nevertheless, the majority of patients described comprehensive care. Some respondents reported having received everything they needed (LEA_2, ASR_3A, ASR_7.2C, ASR_11D), with only very few suspecting that their care did not correspond to that of German citizens (e.g. ASR_21F). Participants reported that providers referred to the government, the duration of treatment or the lack of insurance or employment of ASR as reasons for these restrictions. Some of the affected patients suspected economic reasons (ASR_18E, ASR_22F, ASR_14D). In most cases, the responsibility for access barriers and withheld services was accepted–as communicated by the health professionals–as laying with the government, and was therefore mostly not attributed to the health professionals themselves.

Several participants recognised their legal status and lack of insurance might have led to different treatment, but did not automatically consider this as unfair. For example, ASR_18E stated that care *“is more likely to be offered”* to people with health insurance, since they *“paid for it”*, he recalled sitting in a waiting room and being the next in line, but a German patient was taken first. He explained to himself:

*[…] because she is also working or has paid for it that is now also nothing that is abnormal or causes panic in any way that is all right in my opinion*. *(ASR_18E)*

The occurrence of interpersonal discrimination within the German healthcare system was mostly denied. 12 refugee patients explicitly reported being treated with the same respect and attention as other patients.

*Interviewer: […] Do you have the feeling that you are treated differently in Germany than other people? I mean the experience with medical care*. *Do you have the feeling that you are treated differently in any way?*

*ASR_7.2C*: *Good, very, very well. Respectful. We are very satisfied*.

*ASR_18E*: […] *I think especially the doctors are very correct here they treat everyone the same […].*

Two patients even mentioned that they had expected discrimination or racism, but were positively surprised when they did not encounter it (ASR_2A, ASR_5B):

*[…] I was a bit insecure, maybe even scared at the beginning on the first day in the hospital because I thought I was a stranger*, *but then I saw that I was treated just like every German, just as friendly, just as attentive, yes, and then I was absolutely reassured (ASR_5B).*

Only one participant reported an experience of everyday racism in the hospital, where a nurse reacted dismissively because of a lack of German language proficiency of the refugee patient. This patient was puzzled, because even though she realised that the nurse understood her when she talked in English, the nurse would always respond in German. She recalls that the nurse said: “*Is Deutsch republic*. *Over here speak Deutsch*!*” (ASR_20F)*. The participant describes her feelings in reaction to this interaction:

*LEA_20: Is not good*. *I feel so so so small because is my guilty because I forget something but I [learn] many year and I come in this republic, nobody [invited] me, I come alone and I must learn again to communicate with people who speak Deutsch language but—in my age again learn something is very difficult.*

#### 3.6.2 Assessment of the health system on the basis of previous experiences and expectations

Most participants had a positive overall impression of the German system. We observed that even if some issues had been reflected on critically with respect to individual responsiveness domains, the general overall impression stayed positive. While some participants gave relatively short explanations for their positive assessments (*“Everything is very good” ASR_23F*), others justified their evaluations with the respectful and caring treatment they received in Germany, which sometimes stood in stark contrast to previous experiences: *“They are good*. *They treat me like human*.*” (ASR_1A)*

Some participants noted that this engagement of effort for patients may have differed between physicians and medical contexts. However, the medical competency of doctors was not called into question and a lot of trust was placed in their technical expertise. Many participants noted that the doctors in Germany fulfilled general positive expectations which they pinned to the medical profession:

*About the doctors actually [I can say] only the best because they are doctors and that*’*s what the profession brings with it.” (ASR_18E)*

The experiences of asylum-seeking patients in the German health system need to be contextualised within the wider experiences that participants have had with medical services and health systems both in their country of origin as well as during transit. Expectations shaped through previous experiences prior to arrival in Germany may be a crucial factor in the assessment given to the various domains of responsiveness, as well as the health system overall.

The high socio-demographic heterogeneity of participants meant that previous experiences with medical care varied widely. Some participants remarked that they did not previously receive care for their condition owing to a lack of health insurance and the cost of treatment in the country of origin (ASR_11D, ASR_22F). Other participants spoke of the lack of medical competence and infrastructure in their countries of origin, where their condition could or would not be treated adequately (ASR_5B, ASR_6C, ASR_7.1C). One patient even mentioned serious security concerns regarding seeking more complicated medical care in her country of origin:

*[…] in Fatu when you’re going for an operation it’s the same thing like you’re going to die*. *(ASR_1A)*

Another participant reported that the doctors in his country of origin simply wouldn’t take or have the time to treat his problem adequately. Rather than treating the infection on his leg, they would have amputated it:

*In Antasia you know what they will do to me, they will just cut this my leg*. […]. *My life would be miserable because they cut my leg [and I would] not be able to walk again. In Antasia they cut it. They don’t have time. (ASR_14D)*

In transit countries participants also encountered several barriers to accessing care, including financial or bureaucratic barriers, for example in the form of long waiting times for the approval of treatment by the government: “*They wrote to the government to pay*. *Government never reply*” (ASR_14D). One participant also reported that a doctor in the transit country did not conduct thorough diagnostics, but focused purely on infectious diseases during the medical check-up (ASR_1A).

The German health care system is evaluated against the background of these previous healthcare experiences. Even though we did not specifically prompt participants to recount their patient journeys in the country of origin or during transit, these experiences were often recounted as a way of contextualising the current situation. In some cases, direct comparisons were drawn:

*[…] And here everything is available [In comparison to his country of origin, where he couldn’t afford health care]*. *Here they take care of us.”(ASR_19E)*

## 4. Discussion

The qualitative exploration of the patient journey as experienced by asylum seekers after arrival in Germany provided a rich and varied description of the responsiveness of health services for these patients. Table 2 provides a summary of participants’ experiences of the different responsiveness domains, as well as our assessment of how frequently these resurfaced throughout the patient narratives. Important dimensions of responsiveness, including respectful treatment, communication and trust, resurfaced throughout the narratives of many participants of this study, other dimensions were raised seldom or not thematised and therefore evaluated at all by the participants.

**Table 2:** Evaluation of responsiveness domains emerging in the interviews.

Highly important	**Dignity**	Respectful and caring attitude; taking afflictions and the situation of asylum seekers seriously
	**Communication**	Information easy to understand, attentive communication
	**[Trust]**	Predominantly institutional trust in providers and their expertise
Often evaluated	Prompt attention	Perception of long waiting times, but prompt attention often not expected–humility in the face of the own status of being uninsured
Seldom evaluated	Autonomy	Implicitly thematised, lack of autonomy often accepted and justified by trust in providers
	Provision of Social Needs	Only aspects of a caring attitude of health professionals that exceeded expectations mentioned
	Choice of Provider	Implicitly thematised regarding access barriers to specialists
Not evaluated	Confidentiality	
	Basic Amenities	

Overall, experiences with health services in Germany were portrayed positively. The free and high standard of care patients received often stood in stark contrast to previous experiences. The responsibility of legal care restrictions was seldom attributed to health workers and discrimination rarely reported. Instead, many participants were full of gratitude for the care they *had* received. The efforts made by healthcare staff to be friendly, respectful and take care of their patients, even when acting in restricted circumstances, were recognised regularly. As health service researchers, we attempted to identify barriers, problems and areas for improvement in the care process in this very unique healthcare setting. However, even when particular problems or issues were described, participants were quick to remind us that they were very satisfied with their health care overall, and that they were being taken good care of. It is important to note that this gratitude may reflect the broader situation in which the participants find themselves in. The experience of being physically cared for (given food, shelter and safety) after a potentially long and arduous pre- and peri-migration phase may build the basis of a generalised trust [29] and gratefulness towards German institutions. This observation might also result from social desirability, since interviewees might not want to seem ungrateful, “difficult” or “demanding” to health care providers, researchers, but also to German society, especially while being in this situation of dependency. The resulting humility may be one reason why few expectations and therefore evaluations were observed in some responsiveness dimensions.

The issue of respectful treatment emerged as a particularly important theme throughout patient journey narratives. Positive evaluations of services were often accompanied by descriptions of the friendly and “caring” attitude of medical staff, as well as the feeling of being listened to and taken seriously. However, the high overall opinion of the German health system seems to be mainly connected to the expected and perceived medical competency of doctors, as well as the perception of a system which reacts to (the most pressing) needs of its patients, in contrast to possible previous experiences in other countries. These findings echo previous qualitative research with asylum seekers in Germany, which found that patients deem medical personnel to be competent, friendly and dedicated to ensuring patients have everything they need [22]. The present study confirms these findings while “following” ASR through their patient journey across several sectors, allowing for more nuanced analysis of care provided, starting with the initial health examination, followed by individual care in on-site clinics of the reception centres vis a vis the ambulatory care and the hospital sector.

Throughout all sectors, effective communication was deemed a crucial component of the provision of responsive care from the perspective of ASR patients. This is an issue which has been extensively documented by qualitative research with asylum seekers both in Germany [18, 22, 30] and in Europe [31]. The presented narratives demonstrate the difficulties of finding a translator with the appropriate language competencies and enough time to support communication between physicians and patients. This domain affects asylum seekers differently depending on their language proficiency, as those who speak (some) English or German find it easier to communicate in the healthcare setting. Those needing interpreters often only have access to lay translators, such as other healthcare staff, security personnel or friends and family. Participants express gratitude for this support; issues around confidentiality, trust or shame are not raised with regard to non-professional interpreters. This suggests that there may be a hierarchy among the domains of responsiveness, with some being deemed more important than others. When clear communication with physicians is at stake, issues of confidentiality may be less of a concern. However, the expressed satisfaction with lay interpreters may, again, reflect the desire to be content with the present situation and not placing excessive demands given the precarious circumstances of the asylum situation.

This is the first qualitative study among asylum seekers and refugees which uses patient experiences to understand the responsiveness of care. In fact, the operationalisation of the responsiveness concept with a qualitative methodology is rare, with most empirical assessments choosing a quantitative survey methodology [32]. However, a previous study by Behrensen et al. (2004) covers many of the responsiveness dimensions for ASR in Germany without explicit use of the concept [30]. In their study, waiting times were also described as an issue of high importance for asylum seekers. The authors add that this issue may be particularly pertinent due to the experience of flight, as a result of which many health concerns may not have been addressed for a long time and may thus be particularly urgent. The authors further note that the choice of provider was particularly important to asylum seekers, especially after negative experiences with their initial provider. This stands in contrast to the results of the present study. This may be explained by the fact that participants of the Behrensen et al. study were recruited exclusively from regional accommodation centres linked to regular health care structures, while our sample consisted largely of residents in reception facilities, where choice of provider is largely confined to those working in on-site clinics. Similar to the present study, the analysis by Behrensen et al. shows that a lack of trust in the healthcare interaction and not being taken seriously led to particularly negative experience in the healthcare encounter. The accompanying disinterest and superficial treatment was reported frequently in the study by Behrensen et al. (2004). We also found such accounts, but in a lower frequency. This demonstrates the high heterogeneity of experiences made by asylum seekers in Germany. Further research is needed to understand in which settings or situations this lack of trust or not being taken seriously is particularly evident and how it might be counteracted.

Analysing patient narratives, we found many accounts of high levels of trust in physicians in Germany, despite limited experience with the German medical system. This suggests that we are dealing with “institutional trust” [29], which is conferred to the system and its actors. Positive stereotypes towards the quality of care in Germany and the medical profession appear to be the basis of this trust. Institutional trust in physicians could be observed in reference to their medical training, professional expertise and professional ethical code, obliging physicians to do what is in the patients’ best interest. However, upfront trust was found in some instances to hinder autonomy of decision-making and choice of provider. For instance, opinions expressed by physicians, treatment options and actions taken were not questioned in several situations. In such instances, patients acquiesced to the asymmetric power relationship and did not demand more autonomy.

Continued engagement with a particular healthcare professional or treatment setting allowed for the development of *interpersonal* trust [29] for some participants. Here, trust was fostered through continued experiences of clear communication and respectful treatment in the same healthcare setting or with the same provider. Therefore, in cases where trust in doctors or the healthcare system was not displayed upfront, a trusting relationship could be nurtured through positive healthcare experiences and continuity of care. Both institutional and interpersonal trust continually emerged as important attributes of positive patient experiences throughout the patient narratives. Overall, a trusting relationship between patient and provider emerged both as a key condition for and outcome of a successful health system interaction.

While our results show high overall satisfaction with health services in Germany, using the lens of responsiveness allowed us to identify areas where care can be strengthened further. In particular, the need for a structured and systematic use of interpreters is needed to ensure clear communication and to avoid misinformation or duplication of efforts. Solutions need to be found to ensure adequate translation not only within the outpatient clinics or reception centres, but also with specialist providers and in hospitals. This includes finding sustainable solutions for the provision and financing of interpreting services, which are currently not included in healthcare entitlements and thus provided through short-term or provisional funding arrangements, if provided at all. Investment in the professionalisation of interpreters is also needed, ensuring that enough professionals for relevant languages are available. In some settings, a flexible and practical solution may be to increase the use of tele- or video-interpreting services, as has been implemented in other European countries and some regions of Germany [33, 34].

The fact that some responsiveness domains emerged as more important in patient narratives then others may be influenced by previous experiences with health systems in other parts of the world [22] and particular expectations of what a health system “should” deliver. Some domains are rarely addressed, but this does not necessarily indicate their irrelevance. Rather, it is important to understand why this is the case and whether certain topics—such as autonomy—require special attention precisely because they are rarely mentioned. For example, the World Health Survey showed that individuals from countries with higher education levels and better equipped health systems tend to have higher expectations of and give more value to the autonomy domain [35], although qualitative studies from individual countries are inconclusive about this assessment [36, 37].

Responsiveness refers to legitimate expectations, but many asylum seekers know only roughly which claims they can assert [38]. To foster patient awareness, especially of rarely critically reflected dimensions of their health system encounters, patients could be informed what they legitimately can expect from German providers. The UNHCR offers online sources with information on health care entitlements and confidentiality [39]. This information could be supplemented with findings of our study. ASR patients should know, for example, that professional interpreters are often not provided and given the tools to find high quality interpreters. All patients should be made aware that they can expect to be adequately informed about their diagnosis, treatment, and medicines and that it is their right to make autonomous decisions on this basis.

At the same time, health care providers need to be prepared to protect the interests of their patients, even in cases where patients do not seem to expect and therefore demand it. Health workers should be sensitised to the power asymmetries and dependencies which are intrinsic to treating ASR patients. They should be equipped with conversation techniques to ensure shared decision-making and autonomy of these patients. In general, in the face of the specific challenges in caring for ASR, the health workforce needs more preparation to provide professional healthcare to this patient population. Some specific trainings programs have been developed [40, 41] which foster medical, ethical, pragmatic, transcultural and structural competencies for healthcare professionals caring for ASR patients (27–29). Among their most important goals are increasing awareness of the particular situation that refugee patients are in and the particular context in which their care is situated, which therefore may lead to more empathy and understanding. Given the high importance assigned to being treated respectfully and taken seriously, respect for asylum seeking patients and non-discrimination in the healthcare setting are values which need to be continually reaffirmed in all healthcare settings. Vocational and academic educational institutions should (further) develop training materials and programs regarding ASR care; education policy makers and public health agencies are responsible for ensuring that training materials and courses are accessible nationwide.

From a health system perspective, the continuity of ASR care needs to be improved via an alignment of the asylum and health care processes. Our analysis shows that continued engagement with the health system and its actors tends to lead to improved understanding of system processes as well as increased levels of trust. Given the importance of trust for successful treatment, the continuity of care needs to be supported wherever possible. For example, transfer to other accommodation facilities should be delayed if medically indicated. Information transfer along with patient transfer is crucial. A nationwide implementation of secure patient data management systems that allow data exchange should be fostered by health policy makers.

To ensure high quality care, more research that traces the journey of ASR through the health system and gathers patient experiences is needed. In general, the technical quality of care is well monitored in Germany. Specific institutions are mandated to establish quality standards for health facilities and to monitor their compliance [42, 43]. However, the non-medical quality of care is not monitored in the same way, and only hospitals are obliged “to implement a patient-oriented complaints management system" [43]. Furthermore, existing quality assurance measures are legally anchored within the regulations for statutory health insurance, but newly arrived refugees are uninsured. While care provided by institutions of the general health system, like hospitals or specialists, is subject to the abovementioned quality assurance mechanisms, health care provided within outpatient clinics of refugee “camps” is not covered by these provisions. None of the outpatient-clinics that were visited in the course of this study had an internal quality management system or offered an official complaints procedure. The scientific community can support quality assurance processes by analysing routine data to trace technical quality of care [44], analysing organisational processes for their ability to meet medical and non-medical quality standards [12] and providing empirical insights into patient perspectives. When establishing quality assurance mechanisms for ASR, the linguistic accessibility of processes should be taken into account. In general, national health policy should work towards ensuring that ASR care is subject to the same standards as the care of local populations. International non-governmental organisations should put pressure on governments towards this goal.

### 4.1 Strengths and limitations

This study benefits from the direct, narrative accounts of refugees who were highly diverse in terms of their age, gender, countries of origin, education, health conditions, asylum status, place of accommodation and duration of stay. The research team was comprised of individuals with diverse professional backgrounds and substantial experience in both the setting of refugee reception and health care centres as well as qualitative research. The use of professional video-interpreters allowed for the inclusion of a diverse group of participants. However, in some instances technical problems and the time needed for the translation meant that the conversation could not flow as naturally as it would have without an interpreter. Furthermore, in interviews conducted in languages other than German or English, the analysis was based on a transcription of the translator’s words, which may not always adequately represent the exact wording used by participants. This was part of the reason why content analysis methodology was chosen to avoid an over-reliance on the semantics of translated expressions.

A further limitation was the recruitment of participants through medical staff in outpatient clinics and the researcher’s professional affiliation with healthcare organisations. Even if study objectives and principles of confidentiality were explained to participants in detail in the interview language prior to the start of the interview, participants may have been subject to a positivity bias, feeling that they had to express gratitude for the care received, rather feeling free to criticise it. Due to the recruitment strategy, the patients we talked to were inherently considered as having medically relevant problems by health care staff, which might also explain that these patients felt mostly taken seriously and taken care of. We also focused our recruitment mainly on the German federal state of Baden-Württemberg, with all except two participants living in this state. Despite being one of the largest federal states with substantial regional heterogeneity, we cannot claim to capture the full range of experiences made by ASR patients in Germany. Finally, this study only included the experiences of ASR in reception centres and psychosocial centres. This meant that the majority of participants had only been in Germany for a few months. We cannot adequately capture further stages of the patient journey, when ASR are usually transferred to regional accommodation centres, integrated into regular health care structures and may be dealing with the result of their asylum application. Further research is needed to capture the experiences of ASR during this crucial stage of their journey.

## 5. Conclusion

Asylum seekers and refugees report heterogeneous experiences with health services after arrival in Germany. Against the backdrop of diverse prior experiences with health systems in their countries of origin and migration phase, they give nuanced and diverse assessments of the responsiveness of care in Germany. Respectful treatment, clear communication and trust could be identified as crucial to the delivery of responsive care throughout patient narratives. These factors were prominent reasons given for positive evaluations of the health system, and their absence led to particularly negative experiences. An expansion of high-quality interpreting services, access to professional training to increase the competency of healthcare staff in caring for a diverse patient population, instituting quality assurance mechanisms within ASR care facilities as well as an alignment between healthcare and asylum processes to promote continuity of care could further strengthen the responsiveness of care for asylum seekers in Germany.

## Supporting information

S1 File Attachment 1: Socio-demographic characteristics of participants. (PDF)

S2 File Interview guide. Patient journey ASR.
(PDF)

S3 File Code system. Patient journey ASR.
(PDF)
