# Supplementary material for: The patient journey of newly arrived asylum seekers and responsiveness of care: A qualitative study in Germany
Source: PLoS One. 2022 Jun 24;17(6):e0270419. doi: 10.1371/journal.pone.0270419 (PMC9231813; doi:10.1371/journal.pone.0270419)
Supplement: S1 File — (PDF) [file pone.0270419.s001.pdf]

S1 Attachment 1: Socio-demographic characteristics of participants

**Interviewee**	**Age group**	**Gender**	**Nationality (anonymised)**	**Months since arrival in Germany**	**Inter-viewer**	**Interview language or interpretation**	**Legal status**	**Highest educational attainment**	**Primary health concern/ illness**
ASR_1A[1]	30-39	female	Fatu	2	SZ, FR	English	Asylum seeker	Primary school	Gastro-intestinal complication after surgery
ASR_2A	20-29	female	Fatu	9	SZ, FR	English	Asylum seeker	No formal education	Mental Health Issues
ASR_3A	20-29	female	Fatu	5	SZ, FR	English	Asylum seeker	Vocational training	Myasthenia, Mental Health Issues
ASR_4.1B	40-49	female	Asmait	<1	LB, FR	with interpreter	Asylum seeker	Secondary School	Morbus Crohn
ASR_4.2B	30-39	male	Asmait	<1	LB, FR	with interpreter	Asylum seeker	Secondary School	Diabetes, Hypertension
ASR_5B	40-49	male	Cablait	5	LB, FR	with interpreter	Toleration („Duldung")	Secondary School	Congestive Heart Failure
ASR_6C	30-39	male	Fatu	6	SZ, FR	English	Toleration („Duldung")	Secondary School	Hypertension, Chronic Kidney Disease
ASR_7.1C	50-59	female	Eshijan	3	SZ, FR	with interpreter	Asylum seeker	No formal education	Diabetes, Impaired Mobility
ASR_7.2C	60-69	male	Eshijan	3	SZ, FR	with interpreter	Asylum seeker	Primary school	Inflammatory disease of the jawbone
ASR_8C	30-39	male	Eshijan	1	SZ, FR	with interpreter	Asylum seeker	No formal education	Depression, Chronic Pain
ASR_9C	40-49	male	Udra	11	SZ, FR	with interpreter	Toleration („Duldung")	Secondary School	Mental Health Issues
ASR_10C	20-29	male	Toglos	7	SZ, FR	English	Asylum claim rejected	Secondary School	Tuberculosis
ASR_11D	50-59	male	Eldroa	12	SZ, LB	German	Asylum claim rejected	Secondary School	Depression, Mental Health Issues
ASR_12D	20-29	male	Asnil	6	SZ, LB	English	Asylum seeker	No formal education	Mental Health Issues
ASR_13D	40-49	male	Shoala	2	SZ, LB	with interpreter	Asylum seeker	Vocational training	Mental Health Issues
ASR_14D	30-39	male	Asheau	6	SZ, LB	English	Asylum claim rejected	Secondary School	Skin transplant, Mental Health Issues
ASR_15D	20-29	male	Koflen	7	SZ	English	Toleration („Duldung")	University degree	Knee Pain, Mental Health Issues
ASR_16D	30-39	male	Asheau	15	SZ	English	Asylum seeker	University degree	Vision Impairment
ASR_17D	30-39	male	Naswana	4	SZ	English	Asylum claim rejected	No formal education	HIV, Mental Health Issues
ASR_18E	50-59	male	Asmait	18	LB, FR	with interpreter	Asylum claim rejected	Secondary School	Depression, Hypertension
ASR_19E	40-49	female	Astrana	5	LB, FR	with interpreter	Asylum claim rejected	No formal education	Epilepsy
ASR_20F	60-69	female	Asmait	7	LB, FR	English	Asylum seeker	University degree	Arthritis, Mental Health Issues
ASR_21F	40-49	male	Oshana	11	LB, FR	with interpreter	Asylum claim rejected	Vocational training	Diabetes
ASR_22F	40-49	male	Oshana	8	LB, FR	with interpreter	Asylum seeker	Secondary School	Mental Health Issues
ASR_23F	40-49	male	Oshana	5	LB, FR	with interpreter	Asylum seeker	Secondary School	Diabetes

[1] Upper-case letter following pseudonym indicates place of residency of the ASR at the time of the interview, PSC = psycho social centre interviews

ASR_24PSC	30-39	female	Uspad	84 (7Y)	JB, LF	with interpreter	Asylum seeker	-	Mental Health Issues
ASR_25PSC	30-39	female	Osmiala	27 (3Y)	JB, LF	with interpreter	Asylum seeker	Vocational training	Mental Health Issues
ASR_26.1PSC	30-39	female	Oscada	36 (3Y)	JB, LF	with interpreter	Toleration („Duldung“)	Primary school	Mental Health Issues
ASR_26.2PSC	20-29	female	Oscada	32 M (3Y)	JB, LF	with interpreter	Asylum granted	No formal education	Mental Health Issues
ASR_26.3PSC	30-39	female	Oscada	36 (3Y)	JB, LF	with interpreter	Asylum granted	University degree	Mental Health Issues
ASR_27PSC	40-49	female	Oshana	216 (18Y)	JB, LF	with interpreter	Asylum granted	Primary school	Mental Health Issues
