# Supplementary material for: The patient journey of newly arrived asylum seekers and responsiveness of care: A qualitative study in Germany
Source: PLoS One. 2022 Jun 24;17(6):e0270419. doi: 10.1371/journal.pone.0270419 (PMC9231813; doi:10.1371/journal.pone.0270419)
Supplement: S2 File — Patient journey ASR. (PDF) [file pone.0270419.s002.pdf]

Topic and initial question	Check/Memo
Health and medical history<br><br>**How are you today?**<br><br>**If you think back, can you tell me when and how this started?**	Encourage free narration of experiences, sparingly ask about previous care (oriented towards relevancy for patients)<br><br>• Current state of health<br>• Memories: beginning of current afflictions/pre-existing conditions (before entering Germany)<br>• Afflictions/Conditions (after entering Germany)<br>• Health care in the home country<br>• Health care during the journey<br>• Level of information (disease und care options)<br>• Health seeking migration?
Patient Journey in Germany<br><br>**How did it come about, that you went to see a doctor in germany?**<br><br>**How was that exactly? What happened next? How did it go?**	Encourage free narration, ask in-depth further questions, encourage precise description<br><br>• Background/Back story of first contact with German health care personnel<br>• Patient Journey (step by step)<br>• Experiences with the mandatory health examination (What was it about? What was explained? What examinations were done? What impact did it have on your care/health?)<br>• Stations (Multiple facilities/Outpatient-clinics? Transfer between camps?)<br>• Primary care<br>• Emergency care?<br>• Hospitalisation?<br>• Specialists/Psychologists?<br>• Difficulties/Challenges?<br>• Interfaces/Gate-keepers?<br>• Information transfer?<br>• Financing/Accounting process (health card/paper vouchers)<br>• Knowledge of the health system (Knowledge acquisition?)<br>• Differences to health care in the country of origin?
Health care in the current facility<br><br>**You are currently receiving medical treatment at [name of facility]. How do you experience the treatment here?**<br>**What is good? What is not? What should be changed?**	Experiences, positive/negative evaluation, feelings, personal handling of experiences, wishes for change<br><br>• Receiving information<br>o Consultation hours in outpatient clinic (How and from whom was information provided?)<br>o Diagnosis, medication, therapy<br>• Support/Advice (What kind? From whom?)<br>• Doctor-patient-communication (How? Several different doctors?)<br>• Interpersonal interaction and trust<br>• Language? Barrier and if so: Impact on care?<br>• Information on medications<br>• Role of relatives/Friends/Partner?<br>• Difficulties? Problems?<br>• Costs, withholding of medical measures, care restrictions, dealing with restrictions?

General satisfaction with health care in Germany	
**All in all, how satisfied are you with health care in Germany?**	Free response, specific follow-up questions as needed (e.g., why is „everything good“?)
Specific questions (if topics were not mentioned in the course of the interview)	Topics to be raised and discussed in more detail, if not mentioned by the patients themselves
**I would like to ask you one/some specific question/s…**	• Health condition and health care in the last 12 month<br>○ Residence and condition of accomodation<br>○ Provider contacts<br>○ Difficulties, Problems<br>○ Assessment of health care including explanations<br>• Discrimination (incl. minimal definition: if you are treated differently and disadvantages because of your skin colour, nationality, gender,… or other another reason) – Experiences with discrimination in the health sector?<br>• Availability of an electronic health card? Consequences? Access regulations without card?<br>• Perception of care restrictions?<br>• Out-of-pocket payments for health care?
Final questions	
**Is there anything else you would like to address?**	• Further experience/s<br>• Further topic regarding health care in Germany<br>• Wishes for the future<br>• Suggestions for improvement
