# Supplementary material for: The patient journey of newly arrived asylum seekers and responsiveness of care: A qualitative study in Germany
Source: PLoS One. 2022 Jun 24;17(6):e0270419. doi: 10.1371/journal.pone.0270419 (PMC9231813; doi:10.1371/journal.pone.0270419)
Supplement: S3 File — Patient journey ASR. (PDF) [file pone.0270419.s003.pdf]

S3 Code System

Code system

- **Diseases and afflictions**
  - Mention
  - Course of the disease
  - Knowledge about own disease and therapy (incl. medication)
  - Individual perspective on illness/influence on life/coping strategies
- **Pre-care (home country and escape route)**
- **Escape route reconstruction**
- **Comparisons of health care**
- **Initial examination**
  - Mention/No mention
  - Experience with initial examination
- **Experiences outpatient clinic in the first reception accommodation**
  - Satisfaction / positive evaluation
  - Dissatisfaction / negative evaluation
  - Doctor-patient communication
  - Language barriers / verbal understanding
  - Expectations
  - Other
- **Hospital experience**
  - Dissatisfaction / negative evaluation
  - Satisfaction / positive evaluation
  - Doctor-patient communication & relationship
  - Language barriers / verbal understanding
  - Expectations

Other

Experiences of general practitioners

Dissatisfaction / negative evaluation

Satisfaction / positive evaluation

Doctor-patient communication & relationship

Language barriers/ verbal understanding

Expectations

Other

Experience psychologist/ psychiatrist

Satisfaction / positive evaluation

Dissatisfaction / negative evaluation

Doctor-patient communication & relationship

Language barriers/ verbal understanding

Expectations

Other

Experiences Health care in general

Dissatisfaction / negative evaluation

Satisfaction / positive evaluation

Doctor-patient communication & relationship

Language barriers/ verbal understanding

Expectations

Other

Structure of the health system

Health booklet

Health card / insurance card

Financing

Knowledge about / description of structural factors

Measures withheld

Other

Asylum & Health

Asylum status

Influence of the asylum process on health

Influence of health on the asylum process

Transfer and health, incl. information transfer

Living environment

Social networks

Health Seeking Migration

Dealing with limitations of the asylum process

Other

Discrimination / Differential Treatment

Research Ethics

Wishes for the Future

Other
